# A Randomized Trial to Evaluate the Effect of Local Endometrial Injury on the Clinical Pregnancy Rate of Frozen Embryo Transfer Cycles in Patients With Repeated Implantation Failure 

**Published:** 2016-09

**Authors:** Ensieh Shahrokh-Tehraninejad, Minoo Dashti, Batool Hossein-Rashidi, Elham Azimi-Nekoo, Fedyeh Haghollahi, Vahid Kalantari

**Affiliations:** 1 Reproductive Health Research Center, Tehran University of Medical Sciences, Tehran, Iran; 2 Sint Maarten School of Medicine, American University of Integrative Sciences, Cole Bay, Sint Maarten

**Keywords:** Local Endometrial Injury, Clinical Pregnancy Rate, Repeated Implantation Failure

## Abstract

**Objective:** Repeated implantation failure (RIF) is a condition in which the embryos implantation decreases in the endometrium. So, our aim was to evaluate the effect of local endometrial injury on embryo transfer results.

**Materials and methods:** In this simple randomized clinical trial (RCT), a total of 120 patients were selected. The participants were less than 40 years old, and they are in their minimum two cycles of vitro fertilization (IVF). Patients were divided randomly into two groups of LEI (Local endometrial injury) and a control group (n = 60 in each group). The first group had four small endometrial injuries from anterior, posterior, and lateral uterus walls which were obtained from people who were in 21^th^ day of their previous IVF cycle. The second group was the patients who have not received any intervention.

**Results:** The experimental and control patients were matched in the following factors. Regarding the clinical pregnancy rate, there was no significant difference noted between the experimental and the control group.

**Conclusion:** Local endometrial injury in a preceding cycle does not increase the clinical pregnancy rate in the subsequent FET cycle of patients with repeated implantation failure.

## Introduction

Infertility has been a major problem in human population throughout the history (1), and it is believed to be part of the various medical problems that has increased up to 50% since 1955 in the world and 10-15% of couples who are already suffering from it (2). 

In recent years, Frozen Embryo Transfer (FET) has been recognized as one of the important components of Assisted Reproductive Technologies (ART) (3). Cryopreservation has become a very important procedure in treating infertile couples. Cryopreservation can lower the number of transferred fetuses and risk of multiple-pregnancies (4, 5). Embryo cryopreservation in spite of ovarian hyper stimulation can prominently lower the rate of complications (6, 7). In comparison to other protocols of growth stimulation of several follicles, FET protocols are simpler and their main goals are limited to preparing endometrium for receiving embryo (8).

Although an enormous improvement has been achieved in ART outcomes; (9, 10) the rate of failure of these procedures is very high. And repeated implantation failure (RIF) is a common condition of this method (ART). According to ESHRE report in 2010, only 32.4-33% of IVF transfer cases led to clinical pregnancy (11).

In recent years, the uterus-related parameters have been highlighted for contribution in increased rate of abortions and pregnancy complications including endometrium thickness, low endometrial receptivity and immunological incompatibility (12). Researchers’ interest in these factors is due to importance of the implantation process. Defects in correct implantation is still a problem in the path of achieving satisfactory results in ART which leads to RIF in a lot of patients (13-16). It is said that RIF is due to decreased implantation potential of embryos and endometrium receptivity (14, 15).

In patients with RIF, several methods have been suggested for improving implantation. One of the promising methods is Local Endometrial Injury (LEI) (13, 16- 22). Meanwhile, there are some studies that have reported other results, which do not support this procedure (14, 23- 25).

Overall, there is a controversy going on about LEI, its conditions and time. The aim of the study was to evaluate the effect of local endometrial injury on the clinical pregnancy rate of frozen embryo transfer cycles in patients with repeated implantation failure.

## Materials and methods

In this simple randomized clinical trial (RCT), 120 infertile women who were admitted in infertility clinic of Imam Khomeini Hospital and Infertility center of Shayamehr from January 2013 till December 2014 were evaluated. 

The study was approved by research committee of Valie- Asr Reproductive Health Research Center, Theran University of Medical Sciences and was registered in under IRCT 201311065181N12R2 reference number. Patients were included in the study after being confirmed for study eligibility according to the inclusion criteria such as: age < 40 years, previous history of at least two failure of IVF/ICSI cycles, presence of at least 4 embryos with good quality (grade 1), normal uterus in hysterosalpingography (HSG), sonography, hystrosonography or hysteroscopy, and at least 7mm endometrium thickness at suppository progesterone administration day. Written informed consents were taken from patients. All patients had anatomically normal uterus cavity without any pathology like hyperplasia, malignancy, or endometritis in uterus. No one had received oral contraception agents or GnRH before FET cycle. 

Patients known for the following conditions were excluded from the study: Submucousal, intramural, and subserousalmyoma greater than 5 cm, endometrioma equal to or greater than 3 cm, hydrosalpinx, bilateral obstruction of tube, less than 3-4 embryos, endometrial tuberculosis, previous history of tuberculosis treatment, Asherman’s syndrome, BMI > 30 kg/m^2^, active vaginal or cervical infection, and underlying diseases like diabetes or systemic lupus erythematous.

Patients were evaluated for ovarian cysts by sonography in days 2-3 of the cycle. Sonography was repeated in days 19-21 of their cycle before doing the IVF, and then patients were divided randomly into two ( n=60) in LEI and control groups. Random selection for each method was performed by drawing a piece of printed paper from the plastic bag containing of equal number. Numbers of 1-59 for treatment group and 60-120 for control group were selected and By visiting each patient , randomly a number was out of plastic and according to the number , the group was selected .

In the LEI group, on the day 21 of their cycle before IVF, the group was evaluated for LEI. After this evaluation for LEI, endometrial crashing was done in all 4 uterine walls by moving up and down the PIPELLE in the uterine cavity. Meanwhile, 0.5cc GNRH antagonist (SUPERFACT dose) was administered subcutaneously per day. Patients were asked to refer to clinic on the third day of the next cycle. At the clinic, the SUPERFACT dose was diminished to half and daily 6mg of Estrogen (Estradiol Valerate, Iran Hormone) was started. Patients were followed by Sonography until endometrial thickness reached to 8mm (usually after one weak), and then started 400 microgram (BID) of Progestrone (Suppository Cyclogest). Three days after initiating progesterone, patients underwent FET. All embryo transfers was approved by the highest embryo grading score (embryo more than 8 blastomers with no fragmentation or less than 20 % fragmentation) on day 3, and were performed using the Sydney IVF catheter (k-jets-7019-SIVF; Cook IVF). Thawing were done 2-3 hours before embryo transfer using thawing kit (Kitazato Thawing kit, Japan) on the day of frozen embryo transfer (FET).

In the control group, on the day 21 of their cycle before IVF cycle, 0.5cc GNRH antagonist (SUPERFACT, Germany, Merck) was administered subcutaneously and other steps were the same as LEI group, except undergoing endometrial injury. Patients were followed up to the final outcome of the procedure. 

In this study, clinical pregnancy was defined as visualizing a gestational sac in uterus in week 5 after FET by transvaginal sonography (25). Final outcomes were defined as successfulness or failure of pregnancy. Finally, the study outcome was compared between two groups. Quantitative data were reported as number and percent in the form of mean ± SD. For comparing rate of clinical pregnancy and live birth, chi-square test was used. Quantitative variables were compared with independent t-test between two groups. All analyses were performed by SPSS ver. 16 software. P-value < 0.05 was identified as statistically significant.

## Results

Baseline characteristics as compared between two groups in table 1 showed that there’s no significant difference in any parameter between two groups. Based on both clinical and sonographic examinations, there is not significant difference (p = 0.847) in clinical pregnancies achieved in 21 patients of the LEI group (35%), and 20 patients of the control group (33.3). Table 2 shows the therapeutic outcomes in patients with clinical pregnancy. Stimulation characteristics of the indexed stimulated cycle between the two groups are the same.

According to these findings, rate of live birth and abortion was the same in two groups (p = 0.504).

**Figure 1:**
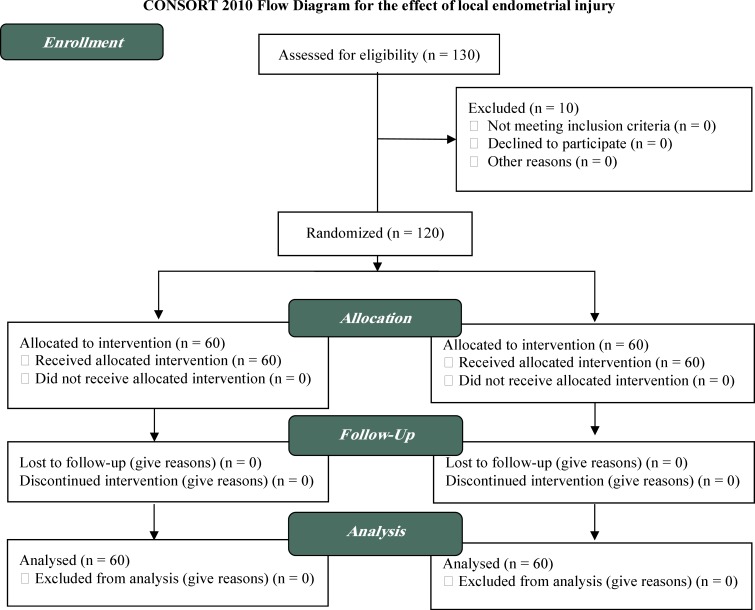
Study flow chart

**Table 1:** Baseline characteristics of two groups

**Group**		**LEI** **(n = 60)**	**Control** **(n = 60)**	**p value**
Age (years) (Mean± SD)		6.4 ± 29.5 (25-40)	5.6 ± 28.3 (25-40)	0.478
BMI) kg/m^2^) (Mean ± SD)		24.8 ±1.7 (22.3-27.6)	25.3 ± 1.3 (23.1-27.3)	0.291
Infertility duration (years) (Mean± SD)		3.2± 6.5 (1-15)	4.3 ± 7.1 (1-16)	0.321
Infertility types (n, %)	Primary	48 (80%)	49 (82%)	0.817
	Secondary	12 (20%)	11 (18%)	0.817
Menstruation status (n, %)	Regular Irregular	33 (55%) 27 (45%)	38 (63%) 22 (36%	0.353
Number of previous IVF cycles (Mean ±SD)		2.3± 0.5 (2-4)	2.8 ±0.7 (2-4)	0.88
Cause of infertility (n, %)	Male factor Female factor Unexplained	4 (6%) 15 (25%) 41(68%)	6 (10%) 16 (26%) 38 (63%)	0.761

## Discussion

Current study show that LEI with PIPELLE before FET cycle cannot affect the outcome and rate of clinical pregnancy and live birth. Local endometrial injury is one of the methods that, by reports, are contributed in increasing success rate of ART. This thought is originated from some experimental studies on animals (26, 27). Recent studies have focused on probable changes in expression of epithelial cell’s genes in injury area (15, 28). KALMA has reported that biopsy can increase membrane proteins’genes’ expression like UROPLAKIN Ib. (28) Although underlying mechanism of increasing success rate of ART with LEI is unknown but generally, related hypotheses are categorized into three main groups: 1.Endometrial injury in previous cycle can induce decidualization which leads to higher probability of implantation (20, 29); 2.Inflammatory response and increase in secretion of cytokines, interleukins, growth factors, dendritic cells and macrophages can all lead to improvements in implantation (30); 3.Endometrial scratching can improve synchronicity of uterus and embryo (19).

The first clinical study regarding the positive effect of LEI in improving results of ART was done in 2003 by Barash et al. These investigators found that endometrial biopsy before IVF can increase the rate of success up to two-fold. They reported that implantation rate (27.7% v. 14.2%), clinical pregnancy (66.7% v. 30.3%), and live birth (48.9% v. 22.5%) were significantly higher in endometrial injury group (16). Thereafter, several studies have been conducted regarding this issue. Zhou et al, performed seven endometrial biopsies from day ten and they figured out that rate of implantation, clinical pregnancy and live birth were higher in the LEI group in comparison to the control group (15).

**Table 2:** Comparison of the IVF-ICSI outcomes in two groups

**Variable**	**LEI** **(n = 60)**	**Control** **(n = 60)**	**p value**
Embryo transfer(n) (Mean ± SD)	3 ± 1.8	3 ±1.2	0.432
endometrial thickness (mm) (Mean ± SD)	3.3 ± 7.2	8 ± 4.1	0.351
Clinical Pregnancy (n, %)			
Abortion	5 (8.3%)	7 (11.7%)	0.457
Live birth	14 (23.3%)	13 (21.6%)	
Ectopic pregnancy	1 (1.7%)	0	
Blighted ovum	1 (1.7%)	0	

In 2010, Gnainsky et al evaluated the role of post-injury inflammation in improving rate of implantation in IVF. Biopsy specimens from endometrium were taken from days 8, 9, 11 and 13. This study showed that inflammatory response can facilitate endometrial preparation for implantation (30). In 2011, Huang et al reported that LEI must be done during IVF cycle to improve outcomes not before initiating the cycle. (19) Another study claimed that LEI doesn’t have any effect on incidence of miscarriage, multiple pregnancies, and the volume and thickness of endometrium (31). In 2009, Li declared that LEI in controlled ovarian hyper stimulation cycle can increase rate of implantation in IVF (20). Nastri et al published a meta-analysis in 2012 in which they reported that LEI done before embryo transfer cycle can improve results of ART, and increases rate of clinical pregnancy and live birth. Note that the LEI must not be done in oocyte retrieval day (21).

On the other hand, local endometrial injury is claimed to be affectless or harmful in some studies. Baum et al conducted a clinical trial on the effect of LEI. They reported that implantation rate in LEI group was lower than control group but the difference wasn’t significantly meaningful. Rate of clinical pregnancy and live birth was lower in the LEI group, too (23). In a retrospective cohort, Dain et al claimed that performing LEI doesn’t have any effect in increasing rate of live birth and clinical pregnancy (24). Recently, Dunne and Taylor made injury to endometrium in luteal phase before FET and found out that chemical and clinical pregnancy rates are the same in the groups with and without injury (25). Karimzade et al in 2012 showed that performing LET in oocyte retrieval day could have a hazardous effect on the results of ART. They figured out that implantation, clinical pregnancy and ongoing pregnancy in LEI group is significantly lower than control group (14).

In 2012, a comprehensive systematic review and meta-analysis of the effect of local endometrial injury in the preceding ovarian stimulation cycle on IVF outcome was assessed (22). The results based on 2062 patients based from seven controlled studies (4 randomized and 3 nonrandomized), suggest that endometrial injury is 70% more likely to result in a clinical pregnancy as opposed to no intervention. In the same year, a Cochrane Library Systematic Review (21) concluded that endometrial injury doubled the chance of pregnancy and live births after IVF treatments. Nevertheless, most of the studies were based on observations of small population samples or non-RCT.

In the current study, we found out that local endometrial injury doesn’t have any effect on rate of clinical pregnancy and live birth. In our study, rate of clinical pregnancy in the LEI group was 35% and in the control group reached 33.3%. Rates of live birth in LEI and control groups are 23.3% and 21.7%, respectively. It is obvious that this technique is not efficacious in improving outcomes. Mustafa Kara study in 2012 showed that Local endometrial injury in the non-transfer cycle increases the implantation and pregnancy rate in the subsequent IVF-ICSI cycle in patients who had previously failed IVF-ICSI outcome. 

The incompatibility between results of previous studies probably originates from differences in design, methods, type of samples of these studies and sample size. Time of scratching and duration of performing procedure in these studies are different, as well as, it is of note that in majority of previous studies, fresh embryos were used; while in our RCT study, we used frozen embryos, which may have an impact on the final outcome. Using PIPELLE in our study was another factor, which may influence final outcome in comparison with other studies undergoing by hysteroscopy.

## Conclusion

Considering the outcomes of both previous studies and this study, we recommend to do more studies on the office-based hysteroscopy to end up with precise results, and also to improve the pregnancies’ outcome in the cases with repeated implantation failure.
